# Reviving Hearts, Restoring Lives: Long-Term Outcomes of Allogeneic iPSC-Cardiomyocytes Transplantation for Advanced Heart Failure Patients

**DOI:** 10.1016/j.jacbts.2025.01.007

**Published:** 2025-02-11

**Authors:** He Zhang, Qian Wang, Xiyu Zhu, Yunxing Xue, Jiaxian Wang, Dongjin Wang

**Affiliations:** a Department of Cardio-Thoracic Surgery, Nanjing Drum Tower Hospital, The Affiliated Hospital of Nanjing University Medical School, Nanjing, Jiangsu, China; b HELP Therapeutics, Nanjing, Jiangsu, China

Heart failure is the leading cause of morbidity and mortality worldwide. Standard treatments are often insufficient to reverse heart failure especially in advanced heart failure patients (NYHA functional class III-IV). Heart transplantation is considered the one last choice, limited by the shortage of donor organs. Cell-based therapies emerge as a promising alternative to restore cardiac function. Human-induced pluripotent stem cells (iPSCs) have the capacity for ex vivo differentiation into highly purified cardiomyocytes, thus making them an ideal source for heart regenerative medicine aimed at restoring lost myocardium.^1^^,^^2^ However, there are still multiple challenges and technical hurdles that need to be overcome, such as large-scale differentiation, optimal delivery route, management of the immune response, long-term safety, and patient stratification.

Here, we report the first-in-human study of direct epicardial transplantation of allogeneic iPSC-derived cardiomyocytes concomitant with open-chest coronary artery bypass grafting (CABG) surgery. The study protocol was approved by the Institutional Ethical Committee of Nanjing Drum Tower Hospital (No. SC2019008), affiliated hospital of Nanjing University.

Two patients with advanced heart failure (NYHA functional class IV) were enrolled and received intramyocardial injections of allogeneic iPSC-derived cardiomyocytes in May 2019.^3^ On completion of the CABG anastomoses, 1 × 10^8^ cardiomyocytes were evenly injected into the peri-infarct tissue at 10 injection sites in the left anterior wall and heart apex. A 1-month immunosuppressant regimen was used, including methylprednisolone, basiliximab, tacrolimus, and mycophenolate mofetil.^4^ Both patients successfully recovered from surgery and completed 48-month follow-ups.

Similar to findings in nonhuman primates’ studies, arrhythmias, including atrial fibrillation and nonsustained ventricular tachycardia, occurred in both patients from day 2 postoperatively. On days 5 to 7, accelerated idiopathic ventricular rhythm started and proceeded to ventricular tachycardia (<130 beats/min) between days 7 to 14. In patients 1 and 2, sinus rhythm returned on day 21 and 33, respectively. Both patients were continuously monitored by electrocardiography, and no ventricular fibrillation was observed. Prophylactic amiodarone was used, and the ventricular arrhythmias did not compromise hemodynamic stability.

**Figure 1:**
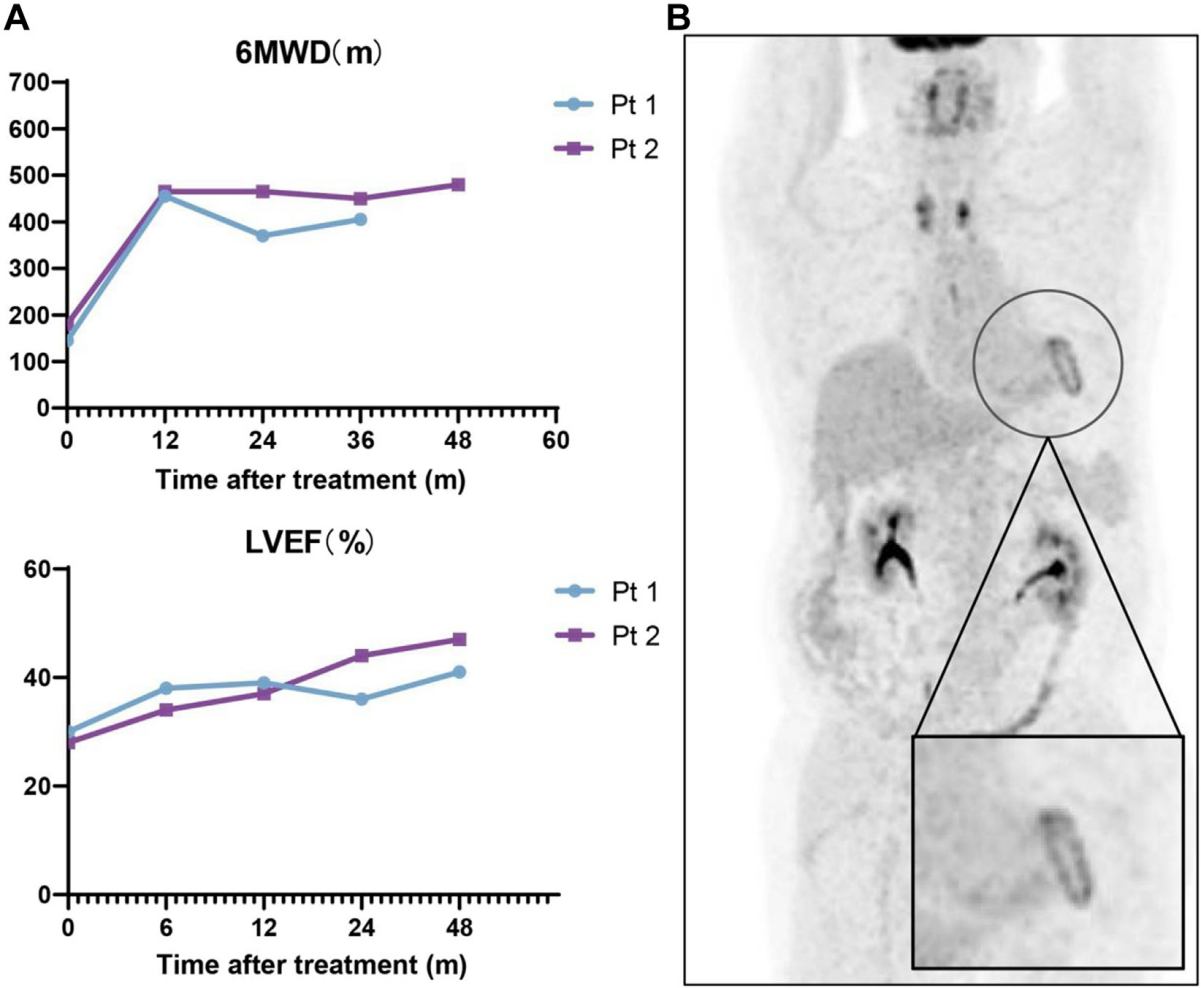
48-Month Outcomes of iPSC-CMs Transplantation (A) Exercise capacity assessments by 6-minute walk distance (6MWD) and changes in left ventricular ejection fraction (LVEF). (B) F-18 fluorodeoxyglucose positron emission tomography at 48 months after HiCM-188 treatment in patient (Pt) 2. iPSCs = induced pluripotent stem cells.

In the current study, exercise capacity (6-minute walk distance [6MWD]), quality of life (QoL), and left ventricular ejection fraction (LVEF) were assessed as potential efficacy endpoints. The 6MWD in patient 1 increased from 145 m at baseline to 455 m at 12 months and remained at 370 and 410 m at 24- and 36-month follow-up. Unfortunately, patient 1 was not able to complete 6MWD at 48-month follow-up because of a stroke. In patient 2, 6MWD increased from an initial 180 m to 465, 465, and 480 m at 12-, 24-, and 48-month follow-ups, respectively. LVEF were also evaluated by echocardiography. Patient 1 had a baseline LVEF of 30%, which increased to 39%, 36%, and 41% at the 12-, 24- and 48-month follow-ups respectively. In patient 2, LVEF increased from 28% pretransplantation to 37%, 44%, and 47% at the same follow-up time points, respectively (Figure 1A).

QoL was assessed by the Minnesota Living with Heart Failure Questionnaire (MLHFQ). Both patients demonstrated an improved QoL, as evidenced by dramatic decreases in MLHFQ scores. MLHFQ of patient 1 decreased from a preoperative score of 74 to 6 (−91.9%) at 1-year follow-up. The improvement of MLHFQ lasted to 36 months and then increased to 49 at 48 months. Patient 2 had a preoperative MLHFQ of 90, which dropped to 7 (−92.2%) at 1-year follow-up and remained low (MLHFQ score: 6) until 48-month follow-up. Heart functions in both patients improved from NYHA functional class IV to II at 12-month follow-up. The NYHA functional class remained at II until the 36-month follow-up and reincreased to NYHA functional class III at 48 months in patient 1, whereas NYHA functional class remained at Ⅱ at 48 months in patient 2.

F-18 fluorodeoxyglucose (^18^F-FDG) positron emission tomography revealed no tumor formation either in heart or in other organs. Surprisingly, perfusion/metabolism of ^18^F-FDG uptake of the left ventricle at 48-month follow-up in patient 2 revealed circular and dot-like ^18^F-FDG uptake enhancements consistent with the site of cell transplantation (Figure 1B), although the confounding effect of the concomitant revascularization cannot be excluded.

In summary, the current first-in-human study of allogeneic iPSC-derived cardiomyocyte transplantation provides the longest safety data in cardiac regenerative therapy and also demonstrates an improvement in heart function. In particular, improvements in exercise capacity, QoL, and NYHA functional class were observed in both patients by 12-month follow-up. No tumors, off-target effects, or severe adverse events were recorded. A striking finding is that the improved cardiac enhancement segment was spatially related to the sites of cell implantation, even at 48 months (patient 2). Although the study design, primarily poised to assess feasibility and safety and which thus entailed a concomitant CABG, prevents distinguishing the effects of cells from those of myocardial revascularization, the clear documentation of safety is a strong incentive to further explore iPSC-derived cardiomyocytes as a new treatment modality for severe heart failure. Further trials, including those under way,^5^ should more specifically aim at addressing the key clinically relevant roadblocks that still need to be overcome: the optimal management of immunosuppression, the efficacy of less invasive routes of cell administration, and the potential benefits of repetitive dosing.
